# Functional Connectivity Pattern Analysis Underlying Neural Oscillation Synchronization during Deception

**DOI:** 10.1155/2019/2684821

**Published:** 2019-02-13

**Authors:** Peng Liu, Hongkui Shen, Shumei Ji

**Affiliations:** ^1^Department of Psychology, Institute of Education Sciences, Shanxi Normal University, Linfen, China; ^2^Department of Biomedical Engineering, Institute of Electrical Engineering, Yanshan University, Qinhuangdao, China; ^3^Network Information Center, Shanxi Normal University, Linfen, China

## Abstract

To characterize system cognitive processes during deception, event-related coherence was computed to investigate the functional connectivity among brain regions underlying neural oscillation synchronization. In this study, 15 participants were randomly assigned to honesty or deception groups and were instructed to tell the truth or lie when facing certain stimuli. Meanwhile, event-related potential signals were recorded using a 64-channel electroencephalography cap. Event-related coherence was computed separately in four frequency bands (delta (1-3.5 Hz), theta (4-7 Hz), alpha (8-13 Hz), and beta (14-30 HZ)) for the long-range intrahemispheric electrode pairs (F3P3, F4P4, F3T7, F4T8, F3O1, and F4O2). The results indicated that deceptive responses elicited greater connectivities in the frontoparietal and frontotemporal networks than in the frontooccipital network. Furthermore, the deception group displayed lower values of coherence in the frontoparietal electrode pairs in the alpha and beta bands than the honesty group. In particular, increased coherence in the delta and theta bands on specific left frontoparietal electrode pairs was observed. Additionally, the deception group exhibited higher values of coherence in the delta band and lower values of coherence in the beta band on the frontotemporal electrode pairs than did the honesty group. These data indicated that the active cognitive processes during deception include changes in ensemble activities between the frontal and parietal/temporal regions.

## 1. Introduction

Studies on the neural basis of deception have important theoretical and clinical implications [1, 2]. Abundant evidence from neuroimaging has revealed how the brain processes deception [3, 4]. Most studies support the hypothesis that greater cognitive control and conflict suppression (i.e., the inhibition of honest reaction) are required during deception [5, 6]. Therefore, in contrast to truthful responses, deception involves the activation of several brain regions, including the temporal and occipital lobes and frontal and parietal scalp regions [2, 7–11]. Furthermore, the prefrontal cortex, including areas of the anterior cingulate cortex, middle frontal gyrus, and inferior frontal gyrus, is usually related to decision-making, action inhibition, and conflict monitoring, which are assumed to regulate deception [3, 12, 13]. At the neural circuit level, the connectivities related to the frontal and parietal networks play crucial roles in the processing of deception [14]. However, compared with high spatial resolution evidence obtained from functional magnetic resonance imaging (fMRI), we focused on the ongoing (i.e., neural oscillatory) components of brain signals using a technique with high temporal resolution because its frequency components have been functionally related to information processing and behavior.

Evidence has indicated that the well-known P300 “oddball” response of event-related potentials (ERPs), i.e., enhanced amplitudes approximately 300 ms following rare, but meaningful, stimuli, may be used as a marker for the detection of concealed information in the “guilty knowledge test” [15]. Consistent with these findings, the increase in the delta band may be related to the response of induced P300 signal detection and decision-making for basic information processing [16]. In addition, Seth et al. [17] reported that the alpha activities exhibited a significant decrease during deception, which could be used to classify truthful and deceptive responses on a trial-by-trial basis. Further, Gao et al. [18] proposed that, at the system level, the connectivities between the prefrontal/frontal and central regions and between the prefrontal/frontal and left parietal regions play crucial roles in the processing of deception. Particularly, high theta synchronization was observed primarily in the abovementioned networks during deception [18]. Thus, the frequency components and high level of synchronization between different regions in a specific frequency band may functionally reflect a transient change in the system process of deception.

Most deception studies to date have investigated activated brain regions, with processes at the system level, particularly those based on neural oscillation, receiving much less attention. As neuroimaging studies have shown that the frontal, temporal, parietal, and occipital scalp regions play an important role in the system process of deception [8, 9, 11], in this study, we hypothesized that a functional connectivity network is formed between activated brain regions that underlie the synchronization of specific neural oscillations involved in the system process of deception.

Emerging studies reported that EEG coherence might be an important method to measure the functional connectivity of specific frequency bands between pairs of cortical regions [18–20]. EEG coherence measures the extent of oscillatory synchronization between two scalp electrode sites' signal within certain frequency bands [21]; the index of EEG coherence is coherence value [22, 23]. The lower (coherence value is 0) and higher (coherence value is 1) levels of coherence reflect the integration of function between two brain areas [24]. Unlike power within specified frequency band indexes the average magnitude of oscillations over a specified time range, EEG measures offer unique information on the strength and synchronization of neuronal activity at high temporal frequencies. Long-distance coherence has been frequently used to survey varying degrees of spatial connectivities [25–29]. Long-range connectivity plays an important role in analyzing the functional connectivity network of the human brain [30] and has been suggested to be associated with higher cognitive functions [31].

In the present study, event-related coherence was used to investigate the functional connectivity between brain regions in specific frequency bands during deception. We aimed to (1) research the functional interactions among neural assemblies distributed across different brain regions during deception and (2) verify the hypothesis that deceptive responses elicit greater connectivity strength than truthful responses, particularly in brain regions specific to neural oscillation synchronization.

## 2. Materials and Methods

### 2.1. Participants

Twenty right-handed students at Yanshan University, China, participated in the experiment. Four participants were excluded due to excessive EEG artifacts, and one participant was excluded due to lack of concentration on the experimental task (no P300 component was found in the target responses for this subject). The remaining fifteen participants (6 men and 9 women; age range: 20-22 years) were included in the final statistical analysis. No participants had a history of neurological or psychiatric diseases. The purpose and experimental procedure were explained to the subjects, and written informed consent was obtained from all subjects. The study was conducted in accordance with the World Medical Association's Declaration of Helsinki for ethical principles for medical research involving human subjects (Declaration of Helsinki 2008) and was reviewed and approved by the Ethics Committee of Yanshan University.

### 2.2. Experimental Procedures

To properly investigate functional connectivity patterns underlying neural oscillation synchronization during deception, participants should first perform a mock crime scenario and then give a deception or honesty response in subsequent tests. Thus, the concealed information test (CIT) [32, 33] was used in this study. The CIT paradigm includes three types of stimuli: (1) the probe (P) stimuli, known only to the guilty individuals, which were related to criminal acts; (2) the target (T) stimuli, known to all subjects, which were not related to criminal acts; and (3) the irrelevant (I) stimuli, unknown to all subjects. The CIT method using P3 components utilizes the bootstrapped amplitude difference (BAD) to identify the deception response. The BAD approach compares the amplitude of P3 responses in P stimuli and I stimuli. For guilty subjects, one expects that the P3 amplitude in P stimuli is greater than in I stimuli. By contrast, for innocent subjects, no difference between P and I is expected, because, for these subjects, the P stimulus is also a type of I stimulus.

Participants were randomly divided into a deception and an honesty group. Six watches and photos of the watches were used in the study. A safe containing two watches was given to the deception group. Each participant was instructed to open the safe and pretend to steal one of the two items. The picture of the stolen watch served as the P stimulus and the other served as the T stimulus. For the honesty group participants, the object (one watch, which was randomly selected from the two watches used in the deception group) contained in the safe was the T stimulus. The photos of the other four watches served as the I stimuli, which were not known by the participants and were not related to the criminal acts.

Participants sat approximately 1 m away from a computer screen in a quiet room. Each session began with a fixation point displayed for 500 ms, after which the stimulus pictures were randomly presented on the screen for 500 ms each. Each stimulus was presented on the computer screen without immediate repetition and with 30 iterations per session. Interstimulus intervals varied randomly from 1.4 to 1.8 s. When the stimulus appeared, participants were instructed to respond by button press as quickly and accurately as possible. All participants in the honesty group honestly responded to the T and I stimuli by pressing the “Yes” or “No” button, whereas the deception group was to deceitfully press the “No” button when the P stimulus appeared. Each session lasted approximately 5 minutes and was followed by a 5-minute rest interval. Each participant performed 5 sessions.

### 2.3. Electrophysiological Recordings

Electroencephalography (EEG) signals were recorded from a 64-channel Ag/AgCl electrode cap mounted on the participant's scalp using NeuroScan Stim2 software (Compumedics, Texas, USA). Electrode positioning in the cap followed the International 10-20 system [34]. Vertical and horizontal electrooculogram (EOG) was recorded by electrodes placed on the infra-/supraorbital ridge of the left eye and 1 cm away from the corners of both the left and right eyes. All electrodes were referenced to the Cz electrode, and an averaged reference was calculated off-line. Impedances at any electrode were kept below 5 KΩ. EEG was continuously recorded with a sample rate of 250 Hz.

### 2.4. EEG Data Processing

Data were analyzed off-line using NeuroScan software. Data were filtered with a band pass in the range of 1-40 Hz. The data were segmented into epochs which contained the data from 100 ms prestimulus to 800 ms poststimulus. The response time was allowed to range from 0 s to 700 ms relative to the stimuli onset. Epochs with a clicking error or a response time > 700 ms were excluded. In addition, to uphold the criterion of ±100 *μ*v, artifacts such as blinks, eye movements, or electrode artifacts were excluded by visual inspection. Finally, twenty-eight P stimulus trials were obtained in each session for each subject, and these trials were used for subsequent analysis. In order to assess the general effect of the experiment, continuous EEGs were locked to the CIT stimuli and the grand averages of the trials within each subject were calculated according to stimulus type. If no P300 component was found in the target responses for a subject, all the experimental data from that subject were excluded.

### 2.5. Coherence

According to previous studies, the frontal, temporal, parietal, and occipital scalp regions were specifically activated during the deception process [6, 9, 11]. Frontal regions are important components of the neural circuits underlying the execution of deceptive actions [12]. Therefore, the frontal (F3, F4), temporal (T7, T8), parietal (P3, P4), and occipital (O1, O2) scalp regions were selected to calculate the coherence for long-range intrahemispheric pairs (F3P3, F3T7, F3O1, F4P4, F4T8, and F4O2) and were further assessed with a particular interest in the connectivities for the delta (1-3.5 Hz), theta (4-7 Hz), alpha (8-13 Hz), and beta (14-30 HZ) frequency bands. The averaged EEG epoch was 900 ms long, and a poststimulus time window of 800 ms was analyzed for coherence. The magnitude of the squared coherence between two channel waveforms, *x* and *y*, was calculated as follows:

(1) $$\begin{array}{c} C_{xy}\left(f\right)=\frac{\left|P_{xy}\;\left(f\right)\right|2}{P_{xx}\left(f\right)P_{yy}\left(f\right)}. \end{array}$$

*C*_*xy*_ corresponds to the magnitude of the squared coherence of the signals *x* and *y* using Welch's average, a modified periodogram method. The magnitude of the squared coherence estimate is a function of frequency with values between 0 and 1 and indicates how well *x* corresponds to *y* at each frequency. The coherence is a function of the power spectral density (*P*_*xx*_ and *P*_*yy*_) of *x* and *y* and the cross-power spectral density (*P*_*xy*_) of *x* and *y*, which were calculated by averaging the 800 ms fast Fourier transforms (FFTs) of the sections of *x* and *y* and the power spectral densities (PSDs) of *x* and *y*, respectively. The coherence analysis was conducted in MATLAB.

### 2.6. Statistical Analysis

In the analysis of long-range functional connectivity in the deception group, the greater connectivity underlying the synchronization of specific neural oscillations was selected according to visual inspection of the long-distance coherence representations for the averaged conditions [21, 35]. In the analysis of intrahemispheric (F3P3, F3T7, F4P4, and F4T8) coherence differences for each frequency band, the group (honesty group, deception group) was the between-subject factor and the band frequency (delta, theta, alpha, and beta) was the within-subject factor. The data were analyzed using two-way repeated measures analysis of variance (ANOVA). Results indicating significant effects were followed with a Bonferroni post hoc test. Values of *p* < 0.05 were considered statistically significant. All data were analyzed with Prism 5 software. Fisher's *Z* transformations were used to normalize the distribution of the average coherence values.

## 3. Results

### 3.1. Long-Range Functional Connectivity Analysis in the Deception Group

**Figure 1:**
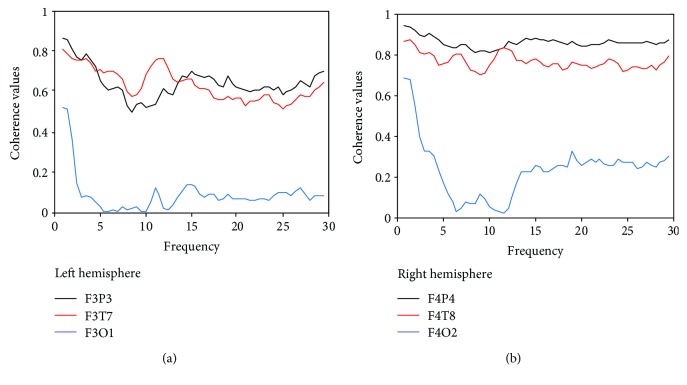
The grand average of evoked coherence during deception. (a) The grand average of evoked coherence for the F3P3, F3T7, and F3O1 electrode pairs in the left hemisphere. (b) The grand average of evoked coherence for the F4P4, F4T8, and F4O2 electrode pairs in the right hemisphere.

To determine the connectivity between the various cortical areas involved in lying, the mean coherence values for the deception group were calculated in four frequency bands.  Figure 1 shows the grand averages of the event-related coherence for the delta, theta, alpha, and beta frequency bands for the left and right hemispheres at the F3P3, F3T7, F3O1, F4P4, F4T8, and F4O2 electrode pairs. In the left hemisphere, the coherence values for electrode pairs F3P3 and F3T7 were higher than those for the electrode pair F3O1 (Figure 1(a)). Similarly, the coherence values of F4P4 and F4T8 displayed a significantly higher value than did F4O2 in the right hemisphere (Figure 1(b)).

**Figure 2:**
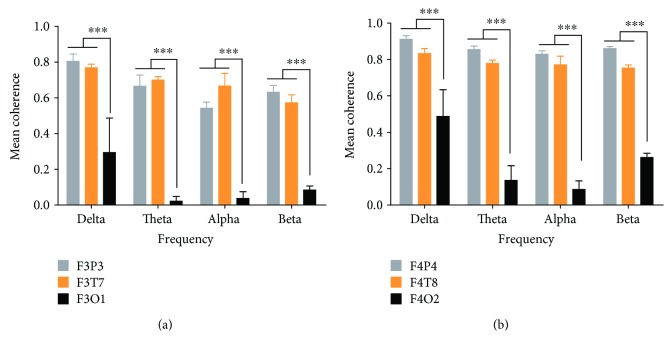
Mean coherence values for deception. Error bars depict standard error mean. (a) The mean coherence for the F3P3, F3T7, and F3O1 electrode pairs in the left hemisphere. (b) The mean coherence for the F4P4, F4T8, and F4O2 electrode pairs in the right hemisphere. ^∗∗∗^*p* < 0.001.

As shown in Figure 2, ANOVA revealed a significant main effect of location (F2, 112 = 2063.29, *p* < 0.001), frequency (F3, 112 = 51.46, *p* < 0.001), and interaction (F6, 112 = 17.47, *p* < 0.001) in the left hemisphere during deception. The post hoc analysis confirmed that the mean coherence values of F3P3 and F3T7were significantly higher than those of F3O1 for the delta (*p* < 0.001), theta (*p* < 0.001), alpha (*p* < 0.001), and beta (*p* < 0.001) frequency bands in the deception group (Figure 2(a)). Correspondingly, ANOVA revealed a significant main effect of location (F2, 112 = 2974.07, *p* < 0.001), frequency (F3, 112 = 66.82, *p* < 0.001), and interaction (F6, 112 = 49.31, *p* < 0.001) in the right hemisphere during deception. The post hoc analysis confirmed that the mean coherence values of F4P4 and F4T8 were significantly higher than those of F4O2 for the delta (*p* < 0.001), theta (*p* < 0.001), alpha (*p* < 0.001), and beta (*p* < 0.001) frequency bands in the deception group (Figure 2(b)). These data indicated that deception induced stronger functional connectivities of the frontoparietal and frontotemporal networks.

### 3.2. Statistical Analysis of Event-Related Coherence for Specific Frequency Bands

**Figure 3:**
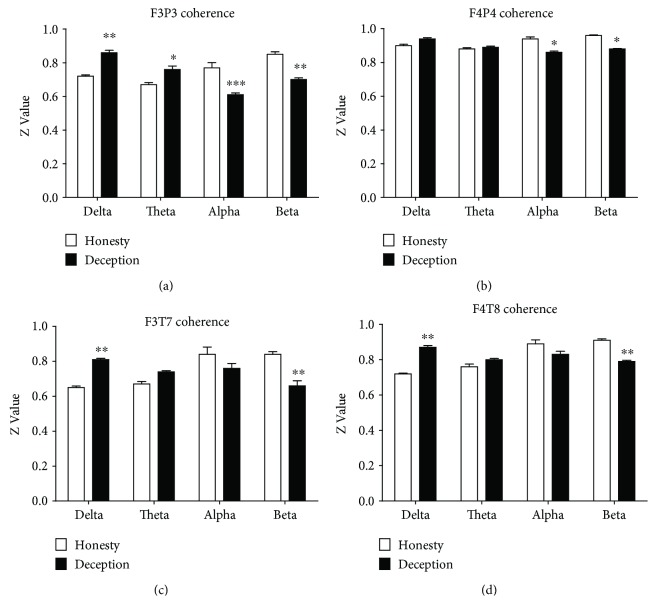
The mean *z* values of the honesty and deception groups for the four frequency bands. (a) The F3P3 electrode pair. (b) The F4P4 electrode pair. (c) The F3T7 electrode pair. (d) The F4T8 electrode pair. ^∗^*p* < 0.05, ^∗∗^*p* < 0.01, and ^∗∗∗^*p* < 0.001.

**Figure 4:**
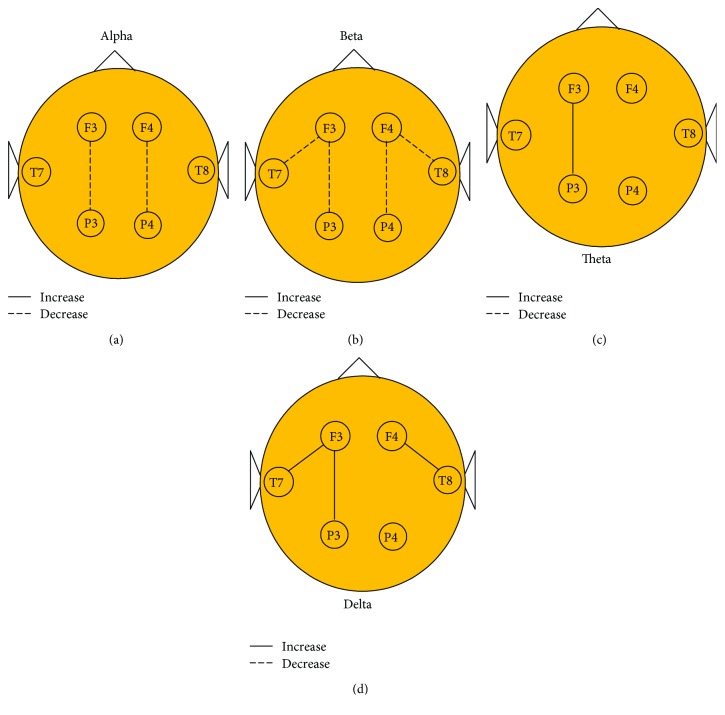
Functional connectivity networks during deception for the four bands (a) alpha band, (b) beta band, (c) theta band, and (c) delta band. The solid/dotted lines represent the increased/decreased connectivities between regions, respectively. The significance of all connections has been corrected by Bonferroni.

Next, we further explored the functional connectivities of frontoparietal and frontotemporal networks in the deception group for the delta, theta, alpha, and beta frequency bands. As shown in Figure 3, ANOVA revealed a significant group × frequency interaction (F3, 39 = 42.81, *p* < 0.001) in the electrode pair F3P3. The post hoc analysis confirmed that the coherence was significantly increased in the deception group compared with the honesty group for the delta (*p* < 0.01) and theta (*p* < 0.05) bands and decreased for the alpha (*p* < 0.001) and beta (*p* < 0.01) frequency bands (Figure 3(a)). Correspondingly, ANOVA revealed a significant main effect of group (F1, 13 = 29.51, *p* < 0.001), frequency (F3, 39 = 11.30, *p* < 0.001), and interaction (F3, 39 = 37.31, *p* < 0.001) in the electrode pair F4P4. The post hoc analysis confirmed that the coherence significantly decreased in the deception group compared with the honesty group for both alpha (*p* < 0.05) and beta (*p* < 0.05) frequency bands (Figure 3(b)). In terms of the connectivity of the frontotemporal brain regions, ANOVA indicated a significant group × frequency interaction in the electrode pair F3T7 (F3, 39 = 23.60, *p* < 0.001) and electrode pair F4T8 (F3, 39 = 42.81, *p* < 0.001). Post hoc analysis confirmed that the coherence significantly increased for the delta (*p* < 0.01) and decreased for beta (*p* < 0.01) frequency bands in the deception group in F3T7 and F4T8, respectively, compared with the honesty group (Figures 3(c) and 3(d)). These results indicate that distinctive roles of frequency oscillations are involved in the frontoparietal and frontotemporal networks during deception (Figure 4).

## 4. Discussion

Our study demonstrated the functional connectivity among brain regions underlying neural oscillation synchronization during deception. We found stronger functional connectivities between the frontal and parietal regions and between the frontal and temporal regions during deception. Furthermore, the deceptive responses elicited a decrease in the connections involving the alpha and beta bands between the frontal and parietal regions compared with the truthful responses. The delta and theta bands increased primarily in the frontoparietal connections of the left hemisphere during deception. Increased delta band activity and decreased beta band activity were induced specifically in the connections between the frontal and temporal regions.

### 4.1. EEG Coherence for the Estimation of Deception-Modulated Functional Connectivity

EEG coherence analysis is an important method to examine the interaction among different brain regions and provides insights into functional network cooperation during various cognitive processes. In the current study, EEG coherence with long-range connectivity analysis, which has been widely used to characterize the correlations among the activities of different neural regions [36, 37] during multiple cognitive processes, including inhibition, set shifting, memory, and conflict monitoring [38], was used to investigate the whole-brain functional connectivity patterns during deception. The identified functional connectivity may shed new light on the neural pattern of deception.

### 4.2. Deception-Modulated Network

In this study, the connectivities related to the frontoparietal and frontotemporal networks were the most discriminating, implying crucial roles of these two networks in the processing of deception.

Accumulating evidence has indicated that the frontal and parietal scalp regions play an important role in the process of lying [2, 8–10, 33, 39]. Frontal regions, especially the anterior cingulate and prefrontal cortices, are important components of the neural circuits underlying the execution of deceptive actions [12]. Previous neuroimaging studies have demonstrated that the anterior cingulate cortex may monitor deceptive responses [40], in addition to being involved in the neurobiology of cognitive control, inhibition responses, and the mediation of conflict, reward, and motivation [41, 42], particularly in decision-making [43–45]. The prefrontal cortex is a critical brain region for complex higher-order task-control functions [7, 46], including enacting plans, rules [47], and strategies [48]. Furthermore, disruption of the dorsolateral prefrontal cortex by rTMS reduces the P300-based marker of deception [49]. Additionally, Johnson-Frey et al. [50] reported that the left parietal cortex is a critical region for the planning of skilled movements, indicating that the parietal regions are related to the execution of deception [50, 51]. Furthermore, ERP-based evidence indicated that P300 waves induced by deception are usually the largest at Pz (the middle parietal scalp region), which strongly indicates the important role of the parietal regions in the deception process [52, 53]. Our investigation demonstrated an increased coherence between the frontal and parietal regions in association with deception. These findings suggest that the frontoparietal networks were synchronously activated during deception and that these regions worked through a cooperative pattern of neural activity.

In this study, the connection between the frontal and temporal lobes was also stronger during deception. Neuroimaging evidence has indicated that the right temporal brain region is activated during deception [54]. Additionally, a P300-based oddball paradigm was generated in the temporal region [55]. The activity of P300 has been associated with the mechanisms of attention allocation and immediate memory processing [6]. Therefore, we speculate that such functional connectivity may be related to the memory information recalled and processed during lie telling. Moreover, our results indicate an extremely weak connection between the frontal and occipital lobes. Combined with the findings of a previous study that showed that the occipital lobes are specifically activated during the deception process [11], our results suggest that neural circuits might not form with the frontal lobe during the regulation of lying.

How did the frontal and parietal/temporal regions communicate with each other to regulate deception? Three possible cooperative patterns of increased coherence between these two networks have been hypothesized [56]. The first hypothesis is that the same generator drives both the frontoparietal and frontotemporal regions. Another hypothesis is that the frontal and parietal regions can mutually drive each other. Similarly, the frontal and temporal regions could be mutually driven by each other during lying. The last hypothesis is that one of the structures (frontal, parietal, or temporal) drives the other structures. Obviously, additional experiments are required to confirm these hypotheses.

### 4.3. Frequency Analysis of Functional Connectivity

For the frontoparietal network, decreased alpha and beta oscillations were produced during deception. Particularly, the mean delta and theta powers were higher during deceptive responses than during truthful responses in the left hemisphere. For the frontotemporal network, increased delta and decreased beta bands were observed during the deception.

There is some evidence to indicate that an increase in the delta power may be associated with attention, particularly in the detection of motivationally salient environmental cues [57, 58]. Another study indicated that the increased delta amplitude during oddball paradigms might be related to signal detection and decision-making [16]. In concordance with these previous studies, our findings suggest that the increased delta band, which primarily involved connections between the left frontal and left parietal regions and between the frontal and temporal regions, may be associated with greater attention resources to the processing of salient conflict. Moreover, increased theta oscillations were reported in our study. Previous evidence has suggested that theta activity generated by frontal regions might be associated with certain cognitive states [59]. A large amount of evidence has linked memory processes with temporal-lobe theta oscillations [60, 61]. However, our results revealed theta synchronization between the left frontal and left parietal regions, rather than within the frontotemporal network. From such a standpoint, the specific association between theta synchronization and the processing of deception, which occurs in the frontal and parietal lobes, remains to be understood. Another explanation of our findings is that the induced theta band increases are strongly linked to the stimulus and are highly consistent with the task [62]. Collectively, the functional role of theta activity during deception is unclear, and further research is required.

Several studies have demonstrated the regulation of alpha oscillations by cognitive functions. For example, increased oscillatory alpha bands were found during high working memory load [63] and allocation attention tasks [64]. Another study indicated that spontaneous and induced alpha oscillations reflect inhibition [65, 66]. Consistent with these results, reduced alpha power may be associated with conflict monitoring and response inhibition [17]. Likewise, Roche et al. [67] proposed that alpha desynchronization might result in a poor performance on a Go/NoGo response inhibition task. Therefore, our results raise the possibility that deceptive responses are involved in the cognitive control of conflict monitoring and response inhibition, which may induce alpha power reduction. That is, the decrease in alpha power in the frontal and parietal regions may be associated with the executive process of response inhibition during deception. An alternative interpretation is that there is a negative correlation between the decrease in alpha power in the frontal and motor cortices. A previous study reported that alpha oscillations might be the primary correlate of the perception of exteroceptive stimuli [68]. Further, the observed increase in alpha has been repeatedly shown in tasks involving visual imagination, especially in visual areas [69, 70]. However, the frontal and parietal lobes are thought to be involved in the executive control of deception [8, 51]. From such a standpoint, this may explain why alpha desynchronization is most prominent in the frontoparietal network.

Our results also demonstrated that the beta band activity decreased during deception in the frontoparietal and frontotemporal networks. The interpretation of this observed decrease in beta power is less straightforward, mostly due to the limited information available related to this frequency band. Previous evidence has indicated that the beta band is sensitive to the discrimination between incongruent and congruent situations [71]. Coherences for frequencies in the band beta during an incongruent situation were significantly stronger than those in a congruent situation in both the left and right hemispheres. However, in our study, neither incongruent nor congruent situations were presented during deception. Therefore, the observed decrease in the beta power in the frontoparietal and frontotemporal networks may actually reflect processing of the incongruent stimulus (e.g., probe stimulus). That is, beta modulation during deception in our study may be related to the mechanisms of conflict inhibition. It is also possible that effective cognitive control may itself require the inhibition of responses to congruent options.

In conclusion, we demonstrated that deceptive responses elicit greater frontoparietal and frontotemporal connectivity strength than truthful responses. Furthermore, our findings indicated the involvement of the alpha and beta bands in frontotemporal connections and the involvement of the delta and beta bands in frontoparietal connections. Thus, we identified new correlates during deception that are complementary to the existing data from electrophysiological and brain imaging studies.

Although the results of our study provide insights into the neural oscillation during deception, there were some limitations to our work. First, only one algorithm was used to assess lying-related EEG. Multiple optimization algorithms for calculation may induce an in-depth analysis of EEG signal and result in a more reliable measure. Hence, developing multiple optimized algorithm calculation into the neural process of lying is our future research goal. Second, although EEG coherence may provide functional integration of two neural populations related to deception cognitive processing, the spatial resolution of EEG is poor. Future studies should combine neuroimaging and neurophysiological methodologies to explore the neural process of deception. Finally, source domain connectivity has more reliable physiological interpretations when using EEG to analyze neural functional connectivity. Obviously, cortical sources of EEG rhythms using source estimation techniques such as eLORETA should be further assessed in future studies.

## Data Availability

The data used to support the findings of this study are available from the corresponding author upon request.
